# Current News

**Published:** 1900-09

**Authors:** 


### Current News.

### HARVARD DENTAL ALUMNI ASSOCIATION.

The Harvard Dental Alumni Association held its Fourth Alumni Day at the school building in Boston, on Monday, June 25, 1900, one hundred and fifty-one persons, aside from patients, being present.

Clinics and demonstrations were given by graduates, and the work of the three classes for the year shown, with many patients present. Essays by four of the professors and instructors were given.

The twenty-ninth annual banquet was held as usual at Young's Hotel in the evening, with one hundred and seven members and guests seated around the festive board.

Mr. Brooker T. Washington, of the Tuskegee Normal and Industrial Institute, Alabama, was the principal speaker, other speakers being Dean Eugene W. Smith, Boston; Mr. Roscoe Conkling Bruce, of Mississippi; Professor Thomas Fillebrown and Dr. L. D. Shepard, of Boston; and Charles W. Rodgers, of the Class of 1900.

The following were elected officers for the ensuing year: Cecil P. Wilson, '72, Boston, President; Henry W. Gillett, '85, Newport, R. I., Vice-President; Waldo E. Boardman, '86, Boston, Secretary; Harry S. Parsons, '92, Boston, Treasurer.

*Executive Committee.*—Waldo E. Boardman, '86; William P. Cooke, '81; Patrick W. Moriarty, '89.

Waldo E. Boardman,
*Secretary.*

Boston, July 2, 1900.

### NEW DENTAL SOCIETY.

The dentists of South Jersey have organized a dental society, with head-quarters in Camden, N. J. It is called the Southern Dental Society of New Jersey.

Monthly meetings are held in the Masonic Temple Building, the third Wednesday evening of each month excepting July and August. The membership numbers twenty-three, and the sessions give unmistakable evidences of the interesting character and virility of the new society. The President is Dr. J. E. Duffield, of Camden; Vice-President, Dr. O. E. Peck, of Bridgeton; Recording Secretary, Dr. A. K. Wood, of Camden; Corresponding Secretary, Dr. W. W. Crate, of Camden; Treasurer, Dr. Mary A. Morrison, of Salem.

*Executive Committee.*—Dr. A. Irwin, Chairman, of Camden; Dr. J. G. Halsey, of Swedesboro; Drs. C. H. Tuttle, E. E. Bower, and A. B. Dewees, of Camden; Dr. J. H. Lummis, of Bridgeton.

---

### NATIONAL DENTAL ASSOCIATION.

Following are the officers of the National Dental Association for the ensuing year: President, G. V. Black, Chicago; Vice-President, West, T. W. Brophy, Chicago; Vice-President, East, E. S. Gaylord, New Haven; Vice-President, South, M. F. Finley, Washington, D. C.; Recording Secretary, A. H. Peck, 92 State Street, Chicago; Corresponding Secretary, Mary E. Gallup, 711 Boylston Street, Boston, Mass.; Treasurer, Henry W. Morgan, Nashville, Tenn.

*Executive Committee* (term expires 1903).—C. N. Johnson, Chicago; V. H. Jackson, New York; T. P. Hinman, Atlanta, Ga.

*Executive Council.*—H. J. Burkhart, Batavia, N. Y.; J. Y. Crawford, Nashville, Tenn.; B. Holly Smith, Baltimore, Md.; W. E. Griswold, Denver, Col.; Thomas Fillebrown, Boston, Mass.

A. H. Peck,
*Secretary.*

---

### VERMONT BOARD OF DENTAL EXAMINERS.

A meeting of the Vermont Board of Dental Examiners will be held at the Pavilion Hotel, Montpelier, Wednesday, October 10, at two o'clock P.M., for the examination of candidates to practise dentistry.

The examinations will be in writing, and include anatomy, physiology, histology, bacteriology, chemistry, metallurgy, pathology, therapeutics, surgery, materia medica, anæsthesia, operative and prosthetic dentistry, together with an operation in the mouth.

Candidates must come prepared with instruments, rubber dam, and gold.

Applications, together with the fee, ten dollars, must be filed with the Secretary on or before October 1.

George F. Cheney,
*Secretary.*

St. Johnsbury.

---

### MISSOURI STATE DENTAL ASSOCIATION.

The Missouri State Dental Association, at their thirty-sixth annual meeting, elected the following officers for the ensuing year: President, F. F. Fletcher, St. Louis; First Vice-President, W. M. Carter, Sedalia; Second Vice-President, F. H. Achelpohe, St. Charles; Corresponding Secretary, B. L. Thorpe, St. Louis; Recording Secretary, H. H. Sullivan, Kansas City; Treasurer, J. T. Fry, Moberly.

*Executive Committee.*—F. M. Fulkerson, Sedalia; W. M. Carter, Sedalia; J. T. Hull, Butler.

*Committee on International Dental Congress during St. Louis World's Fair, 1903.*—William Conrad, B. L. Thorpe, H. J. McKellops, F. F. Fletcher, A. H. Fuller, Walter M. Bartlett, W. F. Lawrenz, all of St. Louis.

Next place of meeting, Sedalia, first Tuesday after July 4, 1901.

---

### HARVARD ODONTOLOGICAL SOCIETY.

At the January meeting of the Harvard Odontological Society the following officers were elected, and were inducted at the February meeting:

President, Dwight M. Clapp, D.M.D.; Recording Secretary, Joseph T. Paul, D.M.D.; Corresponding Secretary, Robert T. Moffatt, D.M.D.; Treasurer, Lyman F. Bigelow, D.M.D.; Editor, Harry W. Haley, D.M.D.

*Executive Committee.*—Joseph T. Paul, D.M.D., Chairman; William P. Cooke, D.M.D.,; Frank T. Taylor, D.M.D.

Robert T. Moffatt,
*Corresponding Secretary.*

---

### FIRST DISTRICT DENTAL SOCIETY OF ILLINOIS.

The eighteenth annual meeting of this Society will be held at Galesburg, Ill., Tuesday and Wednesday, September 25 and 26.

Arthur G. Smith,
*Secretary.*

---

### NORTHEASTERN DENTAL ASSOCIATION.

The sixth annual meeting of the Northeastern Dental Association is to be held at the "Eloise," in the city of Providence, R. I., October 16, 17, and 18, 1900. The Committee has secured the most desirable and commodious building for the meeting ever obtained for such a purpose, and strenuous efforts are being made, with the most gratifying results thus far, to make this meeting the best in every way ever held, at least in New England. Prominent men of the profession have been secured for clinics and papers, the exhibits will be large and so separated from the scientific portion of the meeting that neither will disturb the other. It is earnestly hoped that a large attendance will result.

Edgar O. Kinsman,

*Secretary.*

